# Infective Endocarditis Due to Streptococcus agalactiae Complicated by a Complete Atrioventricular Block

**DOI:** 10.7759/cureus.49676

**Published:** 2023-11-29

**Authors:** Daisuke Miyagishima, Yusuke Ofuka, Aritoshi Kobayashi, Toru Tsujibayashi

**Affiliations:** 1 Internal Medicine, Shizuoka Medical Center, Shimizu, JPN

**Keywords:** perivalvular abscess, complete atrioventricular block, group b streptococcus (gbs), streptococcus agalactiae, infective endocarditis

## Abstract

Infective endocarditis due to *Streptococcus (S.) agalactiae* is an uncommon but potentially life-threatening condition. We report a case of infective endocarditis due to *S. agalactiae* in a 79-year-old woman who presented with fatigue and appetite loss. The results of blood cultures and the vegetation detected by transesophageal echocardiography led us to the diagnosis. She was started on prompt and appropriate antibiotic therapy. Despite her favorable clinical course, she suddenly developed a complete atrioventricular block after one week of conservative treatment. She then underwent surgery with abscess drainage along with aortic and mitral valve replacement. Intraoperative findings revealed that the perivalvular inflammation insidiously extended to the cardiac conduction system and caused a complete heart block. Our case highlights the high virulence of *S. agalactiae*, requiring more vigilance among clinicians.

## Introduction

*Streptococcus (S.) agalactiae*, also known as a group B β-hemolytic streptococcus (GBS), has been recognized as a causative organism for meningitis in newborns and urinary tract infections among pregnant women. However, invasive infections, including primary bacteremia and infective endocarditis, in nonpregnant adults have been increasingly reported [1-5]. Additionally, infective endocarditis due to *S. agalactiae* often causes rapid valve destruction and severe complications, including perivalvular abscess formation and systemic embolization, leading to a high mortality rate. Herein, we report a rare case of infective endocarditis due to *S. agalactiae* accompanied by a complete atrioventricular (AV) block that was successfully treated with appropriate antibiotics and prompt surgery.

## Case presentation

**Figure 1:**
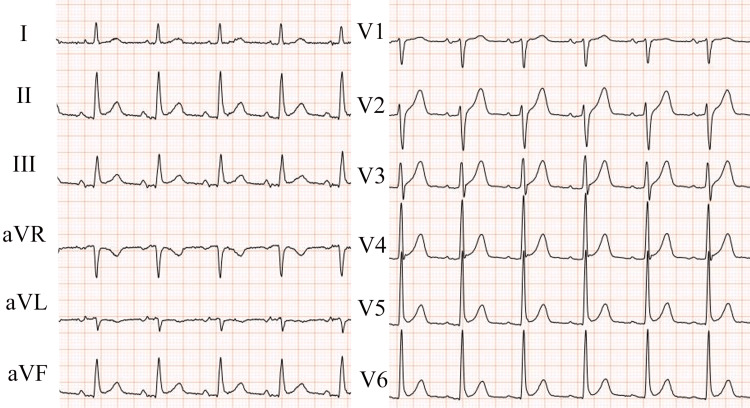
An electrocardiogram on admission. The patient’s electrocardiogram on admission shows a normal sinus rhythm (80 beats/min).

A 79-year-old woman with a five-day history of fatigue and appetite loss was referred to our hospital. Her medical history included hypertension, hyperlipidemia, atrial fibrillation treated with ablation therapy at 50 years of age, and resected uterus cancer 40 years prior. She was taking aspirin, lisinopril, pravastatin, and vonoprazan. On physical examination, she was afebrile (36.4°C) but hypotensive with a blood pressure of 81/39 mmHg and a pulse rate of 90 beats/min. Although she appeared restless and unwell, she was alert and oriented without any focal or sensory deficits. A systolic ejection murmur was heard at the right sternal border. She did not have conjunctival petechiae, splinter hemorrhages, Osler’s nodes, and Janeway lesions. A laboratory examination revealed a high inflammatory state (white blood cell count, 15,500/μL; C-reactive protein level, 19.12 mg/dL; procalcitonin level, 14.33 ng/mL), suggesting the presence of a bacterial infection; however, specific symptoms and imaging abnormalities were undetected. Urine analysis also showed normal findings. Enhanced computed tomography of the chest and abdomen showed unremarkable findings. Moreover, an electrocardiogram (ECG) revealed a normal sinus rhythm (Figure 1). Intravenous fluid therapy was initiated, and her blood pressure returned to the normal range. After obtaining two sets of blood cultures, she was admitted to our hospital for further workup.

**Figure 2:**
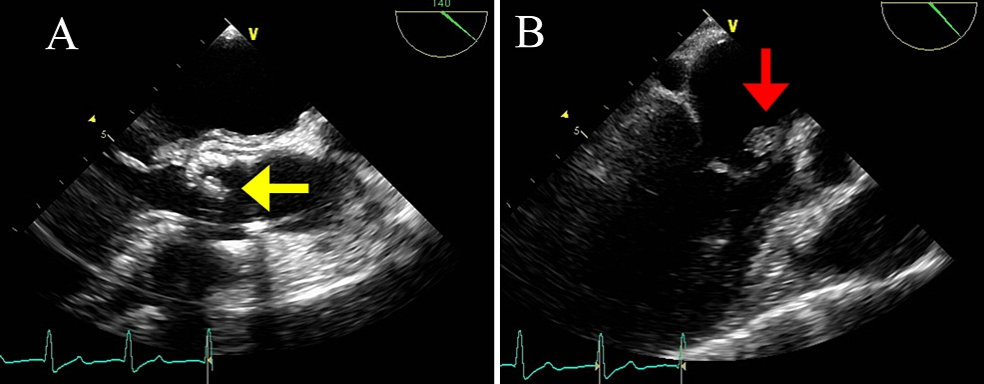
Transesophageal echocardiograms on Day 7 (A) Transesophageal echocardiogram reveals an 8-mm vegetation on the noncoronary cusp of the aortic valve (yellow arrow). (B) A 9-mm vegetation on the anterior cusp of the mitral valve was detected (red arrow).

**Figure 3:**
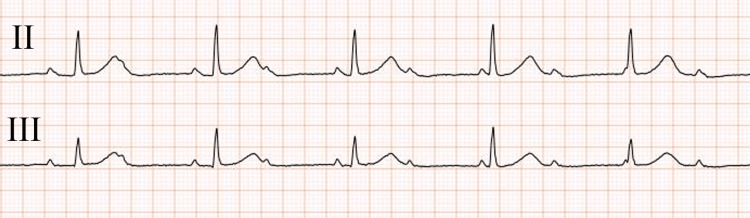
An electrocardiogram on Day 8 The electrocardiogram demonstrates a complete atrioventricular block with a heart rate of 48 beats/min.

**Figure 4:**
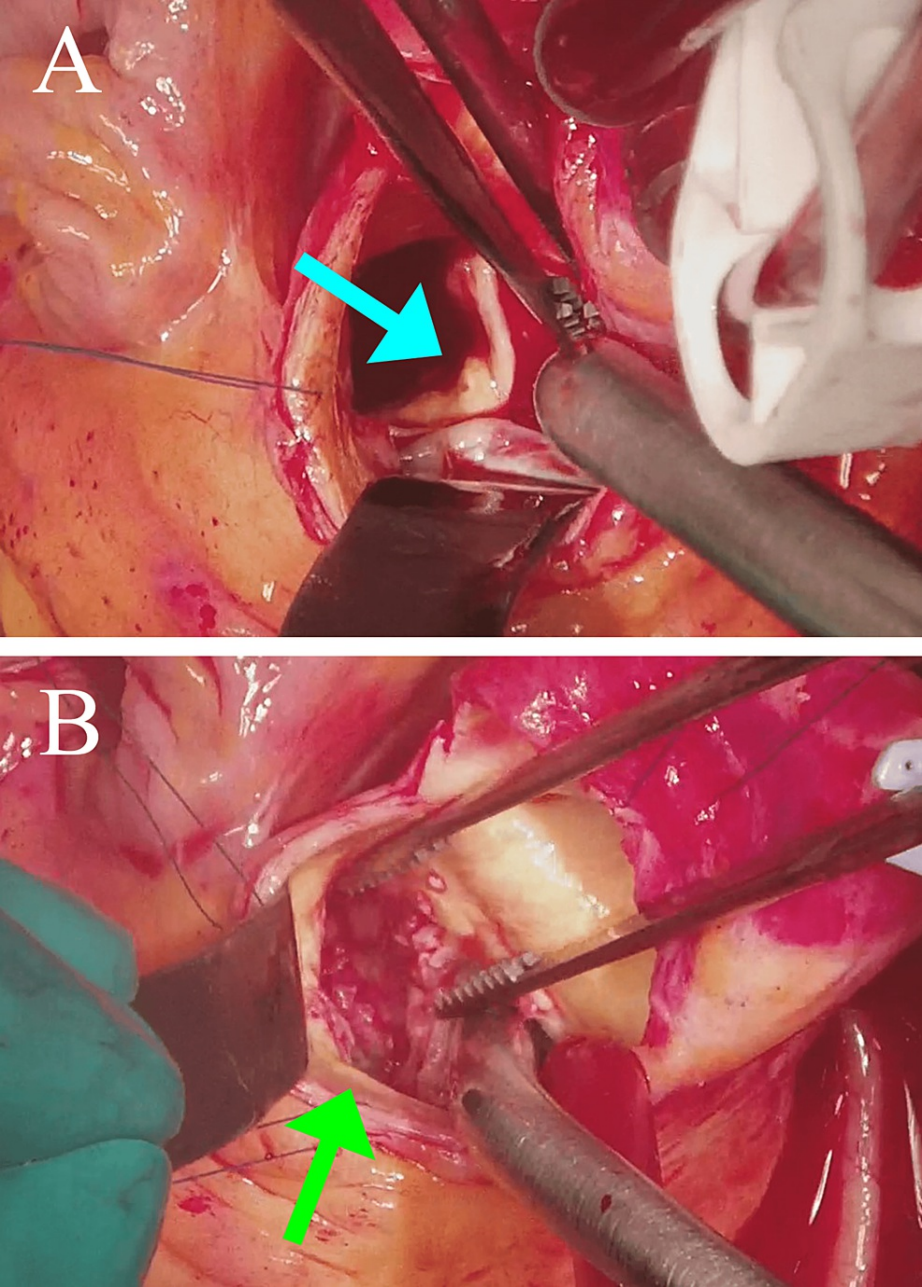
Intraoperative findings on Day 11 (A) The vegetation on the noncoronary cusp was confirmed (blue arrow). (B) A pericardial abscess around the aortic valve was drained (green arrow) and closed with a bovine pericardium.

On the next day, the patient’s temperature rose to 40.1°C and thickening of the noncoronary cusp of the aortic valve, which was consistent with vegetation, was depicted by transthoracic echocardiography; therefore, she was started on an empiric high-dose treatment with ampicillin/sulbactam (12 g/day) and gentamicin (80 mg/day) with a provisional diagnosis of infective endocarditis. She had an ejection fraction of 74% and mild aortic stenosis. On Day 6 of hospitalization, the blood cultures grew *S. agalactiae* and the antibiotic susceptibility testing determined its minimum inhibitory concentration (MIC) of penicillin G to be 0.06 mg/L; therefore, the antibiotics were switched to penicillin G (24,000 U/day) and gentamicin (80 mg/day). Transesophageal echocardiography on Day 7 depicted an 8-mm vegetation on the noncoronary cusp of the aortic valve (Figure 2A, yellow arrow) and a 9-mm vegetation on the anterior cusp of the mitral valve (Figure 2B, red arrow). Based on these findings, a final diagnosis of infective endocarditis due to *S. agalactiae* was established. Given that the patient’s fever improved gradually and her inflammatory indicator levels decreased favorably, conservative treatment with antibiotics was continued. On Day 8, the patient’s heart rate, which had remained at approximately 80 beats/min after hospitalization, decreased sharply to approximately 50 beats/min. An ECG confirmed a complete AV block (Figure 3), and the patient received an urgent temporary cardiac pacing to stabilize her heart rate. Given that a perivalvular abscess was suspected based on the new onset of AV block, a simultaneous aortic and mitral valve replacement was performed on Day 11. Intraoperative findings showed vegetation on the noncoronary and left cusp of the aortic valve (Figure 4A). Additionally, intraoperative transesophageal echocardiography confirmed a perivalvular abscess in the adjacent tissue of the aortic valve, which required abscess drainage (Figure 4B) and patch closure with a bovine pericardium, followed by aortic valve replacement with a bioprosthetic aortic valve. Subsequently, vegetation on the anterior cusp of the mitral valve was confirmed and resected, followed by replacement with a bioprosthetic mitral valve. The patient had an uneventful postoperative course, and her atrial fibrillation returned to a normal sinus rhythm on Day 17 after temporary pacing lead removal. The patient was discharged from our hospital without any complications, and she maintained a sinus rhythm until her six-month follow-up.

## Discussion

Here, we describe a case of infective endocarditis due to *S. agalactiae* in an elderly woman, which was accompanied by a complete heart block and was successfully treated with antibiotics and surgery. *S. agalactiae*, also known as GBS, was previously known as a causative bacterium for meningitis in neonates and for urinary tract infections among pregnant women. Meanwhile, this bacterium has recently emerged as an organism causing soft tissue infections, osteomyelitis, and primary bacteremia in nonpregnant adults [1-5]. Advanced age, uncontrolled diabetes mellitus, obesity, and malignancy are common risk factors for this infection [1-8]. Owing to the aging society, invasive infections caused by this organism are increasing in the United States and Japan [2-4].

Endocarditis due to *S. agalactiae* is an uncommon condition, accounting for only 2.0%-9.1% of whole invasive GBS infections, including bloodstream and soft tissue infections [1-3,8-11]. Contrarily, among all organisms causing infective endocarditis, *S. agalactiae* accounts for only 0.96%-2.6% of cases [9,12-13]. Typically, BGS endocarditis involves the left-sided valves and progresses rapidly, leading to severe valve destruction and complications, including perivalvular abscess formation or systemic embolization. Despite appropriate antimicrobial therapy, many cases still require surgery, and the mortality rate reportedly ranges from 29% to 47% [11-12,14-15].

The frequency of cardiac conduction disorders in infective endocarditis due to *S. agalactiae* reportedly was 10.4% [11]. Once a perivalvular abscess is formed, the inflammation extends to the bundle of His or AV node, resulting in either bundle-branch or AV blocks [16]. A case of infective endocarditis due to *S. agalactiae* that developed a complete AV block has been previously reported [17]. In this case report, the 74-year-old patient could not undergo surgery due to the presence of uncompensated cirrhosis, and postmortem examination demonstrated extensive necrosis of the AV node that had caused the heart block. To the best of our knowledge, this is the second reported case of infective endocarditis due to *S. agalactiae* complicated by a complete AV block. We believe that only immediate surgery can improve the prognosis of patients with infective endocarditis complicated by cardiac conduction defects.

## Conclusions

As cases of primary bacteremia due to *S. agalactiae* in nonpregnant adults have been increasingly reported, the incidence of GBS infective endocarditis with severe complications, such as complete AV block, may also increase. Since GBS endocarditis is more virulent as compared to other streptococcal endocarditis, clinicians should be aware of this rare infectious disease and consider prompt antimicrobial and surgical management to improve the patients’ outcomes.
